# One-Step Immunoassay of Alpha-Fetoprotein Constructed by Silicon-Quantum-Dot-Loaded Porous Gold Nanoshells

**DOI:** 10.3390/nano16080479

**Published:** 2026-04-17

**Authors:** Xiaoling Lu, Chao Shen, You Long, Song Zhang, Fang Chen, Nan Chen, Chenghong Huang

**Affiliations:** 1 School of Chemistry and Chemical Engineering, Chongqing University of Science and Technology, Chongqing 401331, China; 2023205060@cqust.edu.cn (X.L.); 2024205059@cqust.edu.cn (C.S.); 2024205062@cqust.edu.cn (Y.L.); 2025090120@cqust.edu.cn (S.Z.); 2 Chongqing Key Laboratory of Digitalization in Pharmaceutical Process, Chongqing University of Science and Technology, Chongqing 401331, China; chennan0205@cqust.edu.cn

**Keywords:** EHGNs, SiQDs, fluoroimmunoassay, AFP

## Abstract

Alpha-fetoprotein (AFP) is widely utilized for auxiliary diagnosis of primary hepatocellular carcinoma. Therefore, the development of a facile immunosensor is essential for clinical applications. This study aims to develop a simple immunoassay for AFP detection. By incorporating silicon quantum dots (SiQDs) into etching hollow gold nanoshells (EHGNs) via precise nanomanipulation, we designed molecular probes based on SiQDs@EHGNs complex immobilized capture antibodies, which can convert the antigen/antibody binding process into fluorescent divergence signals for AFP measurement. This strategy enabled one-step fluorescence sensing for AFP detection with a linear range of 3.125–200.0 ng/mL and LOD of 0.234 ng/mL. The detection results of 15 clinical serum real samples demonstrated a 93.7% correlation with the market-accepted ECLIA method. The proposed method take advantages of simplicity and rapid response, offering a novel approach for tumor marker analysis with significant potential.

## 1. Introduction

Cancer remains a major global public health challenge in the 21st century [1]. Hepatocellular carcinoma (HCC) is the leading cause of cancer-related deaths worldwide [2]. Featured by low early detection rates, HCC now continues to maintain persistently high incidence and mortality. Therefore, early diagnosis of HCC is crucial. Alpha-fetoprotein (AFP), a critical serum marker in HCC diagnosis [3], is a single-chain glycoprotein with a molecular weight of approximately 70 kDa, containing 3–5% carbohydrates [4]. Liver damage triggers excessive AFP production, elevating serum concentrations. In healthy adults, AFP levels typically remain below 3.4 ng/mL [5], while concentrations exceeding 400 ng·mL^−1^ are clinically considered significant for HCC diagnosis [6]. The development of simple and rapid AFP detection methods holds significant implications. Current AFP detection methods involve electrochemiluminescence immunoassay (ECLIA) [7], enzyme-linked immunosorbent assay (ELISA) [8], radioimmunoassay (RIA) [9], chromatography [10], fluorescence immunoassay (FIA) [11], and electrochemiluminescence immunoassay (ECIA) [12]. While most of these methods basically meet AFP detection requirements, they still have limitations such as dependence on large equipment, lengthy incubation times, use of expensive covalently labeled enzymes or organic fluorophores, susceptibility to environmental interference, poor reproducibility, and even risks of radioactive contamination. Recently, a quantum dot-based fluorescence immunoassay developed by Wang et al. still adheres to the complex operational paradigm of traditional sandwich methods [13]. This approach relies on multiple antibody immobilization, incubation, and washing steps. Such multi-step procedures inevitably increase operational complexity and testing time, limiting their application in rapid screening applications. Therefore, developing a new strategy that avoids multi-step sandwich methods and achieves truly one-step detection is of urgent significance for advancing early diagnosis.

With the advancement of nanotechnology, fluorescent silicon-based nanomaterials have been successfully synthesized and applied across multiple fields. Among these, silicon quantum dots (SiQDs), as a novel class of zero-dimensional nanomaterials, exhibit excellent biocompatibility, ultra-small dimensions, superior optical properties, and water solubility [14,15,16,17]. In recent years, SiQDs have been increasingly utilized to design high-performance fluorescent sensors for chemical or biological analytes [18,19,20]. On the other hand, anisotropic gold nanomaterials, including gold nanostars, nanorods, nanoprisms, nanocages, and nanoshells, have gained great attention due to their outstanding biocompatibility and tunable optical properties [21]. Among various anisotropic gold nanostructures, hollow gold nanoshells (HGNs) stand out with their exceptional optical performance, functionalizable surfaces, low cytotoxicity, and container-like hollow architecture. Not only can HGNs be surface-modified, but they also enable internal cavity loading, providing an ideal platform for spatiotemporal-controlled delivery of drugs or signaling molecules [22]. There have been reports that HGNs reveal remarkable near-field electromagnetic enhancement effects, boosting surface-enhanced Raman scattering (SERS) signals by several orders of magnitude, which enables ultra-low-concentration analyte detection and even single-molecule-level analysis.

**Scheme 1:**
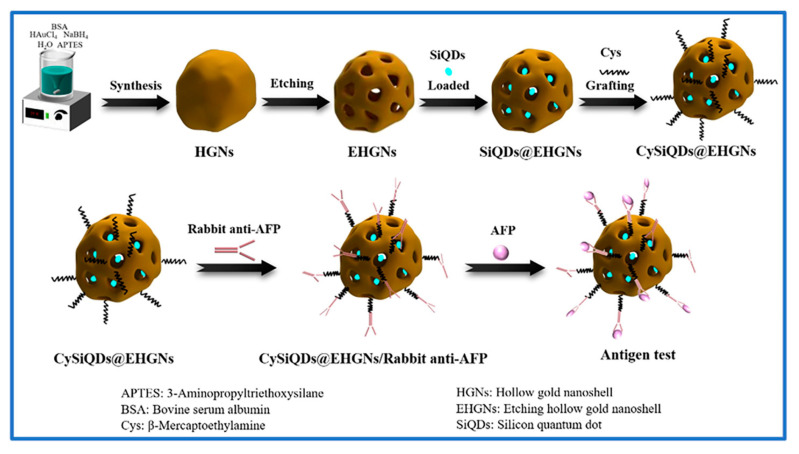
Schematic diagram of a one-step immunoassay method for detection of AFP based on nanocomposite developed by SiQDs incorporated into EHGNs.

Although HGNs possess hollow structures, their internal cavity loading typically requires etching processes to create channels. By delivering SiQDs into EHGNs, this approach not only reduces the impact of surface anisotropy on optical behavior but also effectively shields SiQDs from external environmental interference, thereby significantly enhancing the uniformity and optical stability of the fluorescence signal [23]. Based on the aforementioned analysis, this study designed a novel fluorescent nanocomposite probe (SiQDs@EHGNs). Using etched, porous, hollow, gold nanoshells (EHGNs) as containers, we pioneered the delivery of water-soluble fluorescent SiQDs into their interior cavity. This structure not only effectively shields SiQDs from external environmental interference, enhancing fluorescence signal stability, but more crucially, it establishes the foundation for signal amplification through the localized surface plasmon resonance (LSPR) effect of the HGNs. The probe was externally modified with rabbit anti-albumin (AFP) antibody. During detection, the specific recognition of the AFP antigen by the antibody directly triggers probe aggregation, which significantly amplifies SiQD fluorescence signals via the EHGNs’ plasmon enhancement effect. This mechanism enables detection through a single-step reaction without multi-step incubation. Detailed routes are illustrated in Scheme 1.

## 2. Experimental

### 2.1. Reagents and Apparatus

Ammonium fluoride, 1-(3-dimethylaminopropyl)-3-ethyl carbodiimide (EDC), bovine serum albumin (BSA), triacetoxyethylsilane, allyloxysilane, 3-aminopropyltriethoxysilane (APTES), dimethyl sulphoxide (DMSO), chlorauric acid (HAuCl_4_), hydrofluoric acid, and N-hydroxysuccinimide (NHS) were collectively purchased from China (Aladdin, Shanghai, China) and used without further purification. Hydrofluoric acid (HF), Sodium borohydride, and Span-80 were bought from Kelong Chemical Inc. (Chongqing, China). Alpha-Fetoprotein (AFP) and rabbit anti-AFP antibody were bought from Huayang Zhenglong Biochemical Reagent Inc. (Chengdu, China), and carcinoembryonic antigen (CEA), carbohydrate antigen 19-9 (CA19-9), and human serum albumin (HSA) were entirely gained from KangWei Co., Ltd. (Beijing, China) [24]. Standard AFP was bought from a national institution for the control of pharmaceutical and biological products (Beijing, China) [25]. L-Ascorbic acid (AA), absolute ethyl alcohol, and sodium hydroxide (NaOH) were all purchased from Chuandong Chemical Inc. (Chongqing, China). Sera collected from healthy individuals and patients were immediately stored at −20 °C in a freezer and preserved in our laboratory [26]. A UV–visible spectrophotometer (UV-2600, Shimadzu, Kyoto, Japan), Fourier transform-infrared spectroscopy (FT-IR, Tensor-27, Bruker, Billerica, MA, USA) instrument, X-ray diffraction (XRD, 7000S/L, Shimadzu, Billerica, MA, USA), as well as a freeze dryer (FD-1D-50), Laser granultometer (Mastersizer 3000E, Malvern Panalytical, Malvern, UK), Scanning Electron Microscope (SEM, JSM-7800, JEOL Ltd., Tokyo, Japan), and High-Resolution Transmission Microscope (HRTEM, JEM-2100F, JEOL Ltd., Tokyo, Japan) were equipped in Chong Qing University of Science and Technology. Deionized water (DO) and ultrapure water were supplied in our lab.

### 2.2. Synthesis of HGNs

HGNs were synthesized using the APTES soft template method [27]. In a 50 mL screw-capped glass flask, 9.20 mL of deionized water was added, followed by 20 μL of APTES, and 0.80 mL of 25 mM HAuCl_4_ aqueous solution under magnetic stirring at 300 rpm for 30 s. Subsequently, 0.80 mL of 0.10 M NaBH_4_ aqueous solution was immediately applied, causing the solution to immediately change from yellow to blue-green. Finally, 0.80 mL of 10 mM BSA solution was added, and stirring continued for 1 min to stabilize the HGNs. The resulting HGN dispersion was characterized by UV–vis and TEM analysis.

### 2.3. Preparation of Etching HGNs (EHGNs)

To etch the synthesized HGNs, 2.0 mL of HGN dispersion was first treated with 10 μL of 1 M H_2_O_2_ solution at room temperature for 1 h. Subsequently, 2.0 mL of ammonium fluoride/hydrofluoric acid (NH_4_F/HF) mixture solution [28,29] was added and incubated at room temperature for 2 h to further etch the HGNs. After the reaction, the mixture was centrifuged at 8000 rpm for 5 min, the supernatant was discarded, and the precipitate was collected. Finally, the obtained products were resuspended in an equal volume of DO and later characterized by UV–vis, SEM, and TEM.

### 2.4. Synthesis of Silicon Quantum Dots (SiQDs)

SiQDs were synthesized via a one-step hydrothermal method [30]. The reaction mixture containing 0.5 g of L-ascorbic acid as the reducing agent, 600 μL of triacetoxyethylsilane, and 400 μL of allyloxysilane as silicon sources was dissolved in 4.0 mL of ultrapure water. A total of 5.0 mL of as-prepared solution was transferred to a 25 mL high-pressure reactor lined with polytetrafluoroethylene (PTFE) and heated at 160 °C for 6 h. After cooling to room temperature, the crude product was centrifuged at 8000 rpm for 5 min and filtered through a 0.22 μm membrane to remove large particles. The suspension was then dialyzed for 48 h using a 500 Da molecular weight cut-off dialysis membrane to eliminate small molecular impurities. Finally, the dried SiQDs were stored at 4 °C for subsequent characterization using UV–vis spectroscopy, fluorescence microscopy (FL), and transmission electron microscopy (TEM).

### 2.5. Preparation of SiQDs@EHGNs

The procedure is as follows: 20.0 mL of pre-treated EHGN dispersion was transferred into a 100 mL conical flask, followed by a sequential addition of 2.0 mL of SiQD solution and 5 mL of span-80 solution. The mixed system was stirred at 400 rpm for 60 h at room temperature to facilitate effective SiQDs loading. After reaction, the obtained solution was centrifuged at 6000 rpm for 5 min to collect the SiQDs@EHGNs composite. The supernatant was discarded, and the deposit was purified by ethanol washing. Characterization was performed using UV–vis, fluorescence microscopy (FL) and transmission electron microscopy (TEM).

### 2.6. Immunoassay Process

#### 2.6.1. Preparation of Fluorescent Probes

An amount of 0.50 mL (20 mM) of Cys solution was added to 25.0 mL of SiQDs@EHGNs solution (OD = 0.5), and incubated at 50 °C for 3 h to prepare Cys-modified SiQDs@EHGNs (CySiQDs@EHGNs). Then, 3.0 mL of rabbit anti-AFP (Rabbit anti-AFP, final concentration is 1.0 µg/mL) antibody solution was added to 2 mL of EDC/NHS (mole ratio = 3:1) mixed solution for activation. The activated Rabbit anti-AFP solution was soon added into 23.0 mL of the aforementioned CySiQDs@EHGNs solution and the mixed soluton are incubated for 20 min. The resulting complexes were washed with PBS (pH 7.2) at least two times, as well as 5000 rpm centrifugation to remove the unbound Rabbit anti-AFP. Thus, CySiQDs@EHGNs-labeled rabbit anti-AFP capture probe was successfully prepared after 1.0 μg/mL BSA blocking.

#### 2.6.2. Immunoassay Procedure

Diluted AFP antigen solution was added to the capture probe solution and incubated at room temperature for 120 min. The capture antibody specifically catches the corresponding antigen, AFP, forming an antigen/antibody complex. After the reaction, the mixture should still be rinsed with PBS at least twice to remove excess AFP. FLD and UV–vis spectroscopy were used to analyze the signal response. The probe without AFP addition was used as a blank control, and the antigen–antibody complexes obtained after adding different concentrations of AFP antigen served as positive reactions. Fluorescence response and UV response signals were measured, respectively, according to the specifications of the facilities.

#### 2.6.3. Calibration Curve, Repeatability, Accuracy and Specificity

Under the optimized conditions, application of AFP (800.0 ng/mL, 700.00 ng/mL, 600.0 ng/mL, 500.0 ng/mL, 400.0 ng/mL, 300.0 ng/mL, 200.0 ng/mL, 100.0 ng/mL, 50.0 ng/mL, 25 ng/mL, 12.5 ng/mL, 6.25 ng/mL, 3.125 ng/mL) occurred to establish a dose–responsive curve and calibration curve. The lower limit of detection (LOD) for this immunoassay was determined by the following procedure: First, three zero standard samples were measured for fluorescence signals, and their mean value was calculated. The mean value was then increased by three times the standard deviation (SD), and the corresponding concentration was calculated by combining with the standard curve. Repeatability was evaluated using the coefficient of variation (CV); five independent measurements were performed on standard samples at concentrations of 25.0 ng/mL, 50.0 ng/mL, and 100.0 ng/mL, with corresponding averages calculated. Accuracy was validated through a spiked recovery experiment; known concentrations of the AFP standard were added to predetermined serum samples, and the recovery rate was measured and calculated to evaluate the analytical method. A specificity assessment was conducted through comparative analysis of fluorescence signal intensities under identical experimental conditions using blank controls, positive controls (100.0 ng/mL AFP), and multiple interfering substances, including turbid solution (carcinoembryonic antigen (CEA), carbohydrate antigen 199 (CA199), human serum albumin (HSA), commercial milk, hemolytic serum, glucose (3.9 mM), and inorganic salt K^+^ (3.5 mM), Zn^2+^ (62 µM), Mg^2+^ (1.12 µM), Ca^2+^ (2.25 mM), Na^+^ (135 mM), Cu^2+^ (21.8 µM), Fe^2+^ (7.52 mM), and PBS buffer (0.1 M)).

### 2.7. Methodology Comparison

Serum samples from healthy individuals and patients with primary liver cancer (n = 15) were collected and stored at −20 °C until testing. Prior to analysis, all samples were centrifuged. The recovered supernatant (50 μL) of each sample was diluted by 4 mL PBS (pH 7.2). The prepared SiQDs@EHGNs/Rabbit anti-AFP capture probe was mixed with each diluted serum sample at a 1:1 volume ratio to achieve a final reaction volume of 4.0 mL. The mixture was incubated at 37 °C for 1 h to promote antigen–antibody binding, forming antiAFP/SiQDs@EHGNs capture antibody-AFP in serum complexes. Characterization of these complexes was performed using FL spectroscopy or UV spectroscopy. Each sample was measured at least three times in parallel, and the average value was calculated based on a pre-established calibration curve.

## 3. Results and Discussions

### 3.1. Synthesis of SiQDs

**Figure 1:**
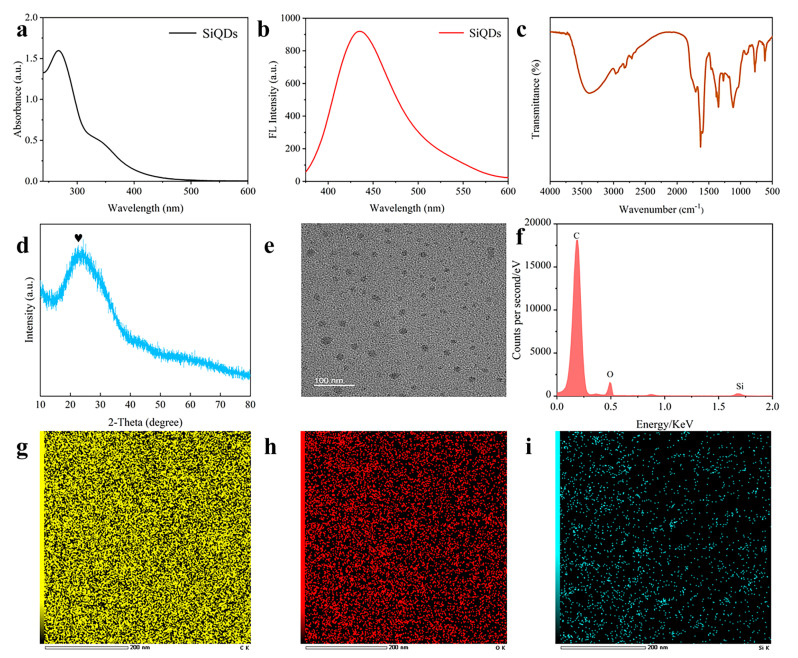
UV spectrum (**a**), FLD spectrum (**b**), FT-IR spectrum (**c**), XRD pattern ((**d**), the peak is heart-shape symbol marked), TEM image (**e**), energy dispersive spectrum (**f**), C spectrum (**g**), O spectrum (**h**), and Si spectrum (**i**) of SiQDs.

The ultraviolet–vis absorption spectrum of SiQDs exhibits a distinct characteristic absorption peak at 269 nm (Figure 1a). The FL spectrum displays optimal excitation wavelength at 340 nm; the maximum emission wavelength of SiQDs is observed at 430 nm (Figure 1b). FT-IR spectroscopy revealed chemical bonds of surface groups from SiQDs (Figure 1c). The broad absorption peak at 3380 cm^−1^ corresponds to O-H stretching vibrations, while the absorption peak at 1705 cm^−1^ is attributed to C=O stretching vibrations. Peaks at 1382 cm^−1^ and 1272 cm^−1^ originate from C-O stretching vibrations, and the characteristic peak at 773 cm^−1^ indicates Si-C stretching vibrations. Surface functional groups, such as-COOH and-OH, confer excellent water solubility and stability to SiQDs. The XRD pattern revealed a diffraction peak at 2θ = 23° of SiQDs (Figure 1d) [31]. The TEM image (Figure 1e) demonstrates that the prepared SiQDs exhibit excellent dispersion, with spheroidal morphology and an average particle size of approximately 2 nm [32]. EDS energy dispersive spectroscopy analysis (Figure 1f) and corresponding elemental distribution maps (Figure 1g–i) indicate that the SiQDs are primarily composed of carbon (C), oxygen (O), and silicon (Si) elements.

### 3.2. Synthesis of HGNs and Preparation of EHGNs

**Figure 2:**
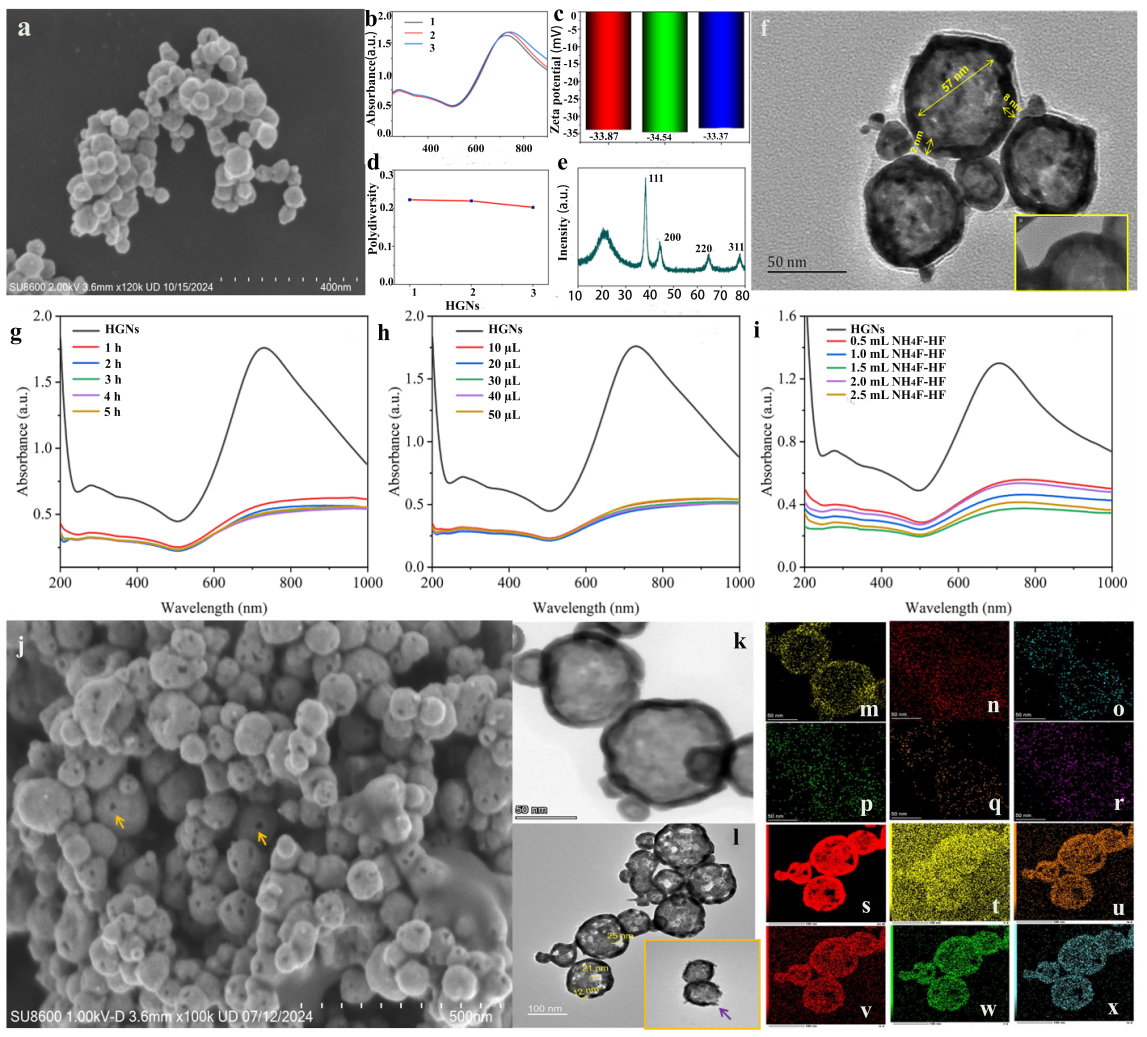
SEM image of synthesized HGNs (**a**), ultraviolet absorption spectrum of HGNs (three batches of duplicate samples) (**b**), Zeta potential of HGNs (three batches of duplicate samples) (**c**), Polydispersity of HGNs (three batches of duplicate samples) (**d**), XRD of HGNs (**e**), TEM of HGNs ((**f**), the yellow boxed area clearly reveals the thin-shell structure of HGNs; the yellow arrows indicate its diameter and shell thickness, respectively), ultraviolet absorption spectra of HGNs etched by 20 µL H_2_O_2_ and 0.2 mL NH_4_F/HF with different times (**g**), ultraviolet absorption spectra of HGNs etched by H_2_O_2_ (10 μL, 20 μL, 30 μL, 40 μL, 50 μL) and 0.2 mL NH_4_F/HF (**h**), ultraviolet absorption spectra of HGNs etched by 20 µL H_2_O_2_ and different volumes (0.5 mL, 1.0 mL, 1.5 mL, 2.0 mL, 2.5 mL) of NH_4_F/HF solution (**i**), SEM image of EHGNs, The yellow arrow indicates the nanopores on the 263 surface of EHGNs (**j**), TEM image of native HGNs (**k**), TEM image of EHGNs with etching indentations are sown by purple arrow in inset (**l**), elemental composition of HGNs (**m**–**r**) and EHGNs (**s**–**x**).

Synthesis of HGNs using APTES as a soft template primarily relies on the stretching amino active sites of APTES in water to stay trivalent gold ions via coordination interaction, hence these ions are evenly distributed across the APTES micelle surface [22]. Upon addition of a reducing agent, the adsorbed trivalent gold ions are reduced to zero-valent gold atoms so that they do nucleate and gradually grow on the APTES surface to form a continuous gold shell. The obtained HGNs solution looks ink-green in color and exhibits a maximum plasma absorption peak at 700 nm. Figure 2a shows a SEM image of original HGNs. The HGNs exhibit LSPR effects and display a characteristic ultraviolet peak at a wavelength of 755 nm (Figure 2b). The average potential value measured for three batches of HGNs was −33.4 mV (Figure 2c). The polydispersity index (PDI) value within the range of 0.20–0.22 is below the narrow distribution threshold of 0.3, indicating that the obtained HGNs exhibit a monodisperse state (Figure 2d). The XRD results (Figure 2e) show that the peak at 2θ = 38.3° corresponds to the (111) crystal plane of the face-centered cubic structure of gold, the peak at 2θ = 44.3° corresponds to the (200) crystal plane, and the diffraction peak at 2θ = 64.7° corresponds to the (220) crystal plane of gold. The intensity of these peaks is related to factors such as the crystallinity and particle size of the HGNs. Additionally, a diffraction peak at 2θ = 77.9° is observed corresponding to the (311) crystal plane [27]. The TEM image in Figure 2f displays a typical core–shell structure with bright centers and dark edges. During the synthesis of HGNs, partial APTES, used as a soft template, undergoes hydrolysis to form unstable silanol compounds. Under specific conditions, these silanol groups undergo dehydration–condensation reactions to form Si-O-Si bonds. As the condensation reaction progresses, the generated Si-O-Si bonds continuously accumulate and interconnect, ultimately forming a three-dimensional SiO_2_ network structure [33,34]. The high electron density of the outer layer corresponds to a dense gold shell (the inset in Figure 2f), while the bright core in the low-contrast region represents silicic residues formed by APTES hydrolysis.

To etch ideal nanopores on the HGN surface, we employed a dual-etching scheme using H_2_O_2_ solution [35] and NH_4_F/HF buffer solution [28,29]. Optimization experiments demonstrated that the combination of 20 μL of H_2_O_2_ (1 M) and 2.0 mL of NH_4_F/HF (V_40% NH4F_/V_40%HF_ = 6:1) for 2 h etching can achieve an ideal nanopore on the HGN surface [36]. The maximum absorption wavelength of the product shifted from 815 nm to 843 nm (Figure 2g–i), with well-preserved UV characteristic peaks of HGNs. The SEM (Figure 2j) and TEM images (Figure 2l) obtained after H_2_O_2_@NH_4_F/HF co-etching reveal significant erosion defects on the gold shell with clearer cavity contours, confirming effective silicon core removal that is accompanied by nanopore formation on the gold shell, with pore sizes of approximately 12–25 nm (yellow arrow illustrated by inset of Figure 2j, and purple arrow revealed by inset in Figure 2l). To analyze elemental composition and distribution changes before and after etching, EDS surface scanning analysis was performed of the HGNs and EHGNs. The results indicate that both constituents primarily consist of Au, C, N, O, and Si elements (Figure 2m–r and Figure 2s–x are, respectively, attributed to HGNs and EHGNs). The trace F signal was also determined from HGNs (Figure 2q). It might be result from spectral line overlap or background interference caused by O element characteristic peaks with adjacent F element peaks during EDS testing rather than actual fluorine content [37]. Post-etching Au element energy spectra (Figure 2w) show distinct voids and discontinuous regions, which precisely correspond to the gold shell loss areas observed in the TEM images [38]. This morphology-component correlation confirms that co-etching simultaneously removes silicon templates while selectively etching gold shells, successfully constructing porous EHGNs.

### 3.3. Construction of SiQDs@EHGNs Fluorescent Probe

**Figure 3:**
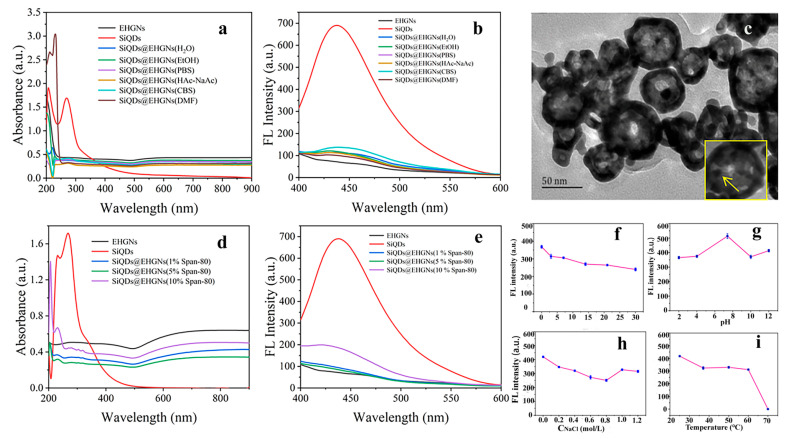
Ultraviolet absorption (**a**,**d**), FL intensity (**b**,**e**) of EHGNs, SiQDs, and SiQDs@EHGNs in H_2_O, EtOH, PBS, HAc-NaAc, CBS, and DMF solutions, as well as in 1%, 5%, and 10% Span-80 solutions. TEM image of SiQDs@EHGNs complex (**c**), and stability of long-time (**f**), ionic strength (**g**), pH (**h**), and temperature (**i**).

We herein prepare a fluorescent probe by loading SiQDs into the interior of EHGNs. The first consideration is the size match between SiQDs and the nanopore on the EHGN surface. The problem was faultlessly solved by adjusting the appropriate size (>10 nm) of the etched hole to accommodate SiQDs (about 5–10 nm) via nanomanipulation; and then there is still the consideration of the blocking effect related to the chemical properties of nanopores, such as charge, polarity, and non-specific interactions when SiQDs were tethered at the gate of the nanopores. To achieve efficient loading, we first systematically investigated the impact of the solvent type on assembling. The UV characteristic absorption peaks of EHGNs exhibit blue-shift using H_2_O, EtOH, PBS, and CBS as solvents while express red-shift for HAc-NaAc and DMF. Characteristic absorption peaks at 230 nm appear in the presence of H_2_O, CBS and HAc-NaAc, but the strongest intensity recorded from DMF . The surface state absorption (260 nm) of the SiQDs weakened, while the core bandgap absorption (206 nm) showed relative enhancement, likely due to SiO_2_ layer formation by air oxidation. Over time, SiQDs may undergo aggregation or dissolution, resulting in size distribution changes. If dispersed in solvents, variations of the absorption spectrum could arise from solvent evaporation or degradation, which is presumably attributed to solvent effects. As illustrated in Figure 3b, the relatively high fluorescence intensity of SiQDs/EHGNs with CBS solvent ensures it was selected as the preferred solvent. We further adopted span-80 (sorbitol monooleate, hydrophile-lipophile balance value is 4.3) to adsorb on the surface of SiQDs to dissociate them, which was evaluated by ultraviolet absorption (Figure 3d) and fluorescence emission efficiency (Figure 3e). The well-dispersed SiQDs can easily enter the interior of EHGNs, as span-80 will also anchor onto the inner surface of EHGNs. At the same time, this arrangement generates steric hindrance effects that effectively suppress the aggregation and structural collapse of EHGNs, thereby maintaining the integrity of their porous structure, offering a flexible tool to regulate interactions between SiQDs and channels, and reducing unnecessary repulsion or attraction. The as-prepared SiQDs@EHGNs composite (Figure 3c) maintained enough FL signal for later use, although there was a slight blue shift at 426 nm compared to that at 423 nm of SiQDs, indicating that EHGNs restrict photoluminescence performance of SiQDs by influencing surface functional groups due to the quenching effect [30]. A stability test using fluorescence intensity measurement demonstrated that the SiQD/EHGN composite exhibits long-time stability (Figure 3f), broad pH tolerance (Figure 3g), and high salt concentration tolerance (Figure 3h), but undergoes thermal decomposition when temperatures exceed 60 °C (Figure 3i).

### 3.4. Establishment of Immunoassay

**Figure 4:**
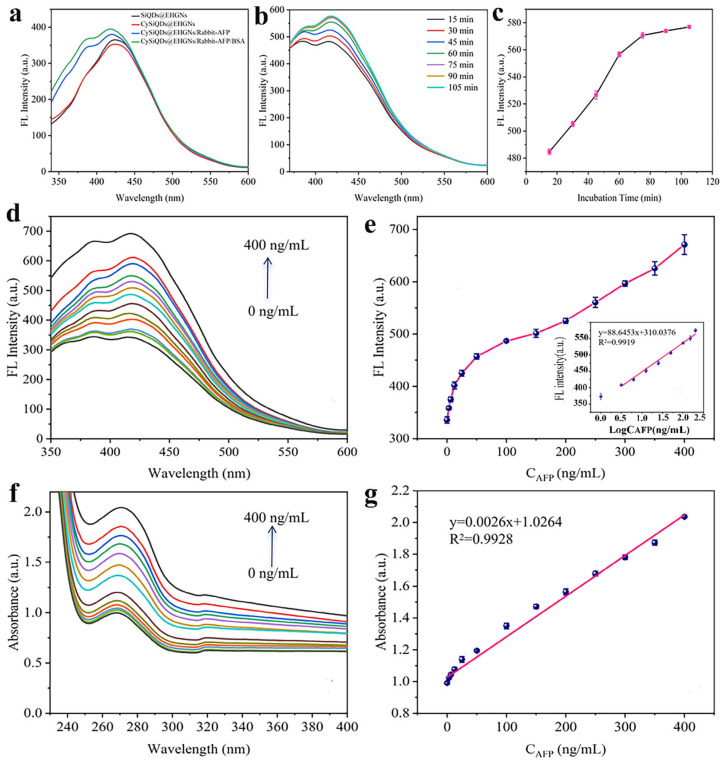
FL signals of antiAFP immobilization on CySiQDs@EHGNs via conjugation between antiAFP with initially coupled cysteine (Cy) by EDC/NHS activation (**a**), fluorescence intensity with different incubation times of AFP and antiAFP/SiQDs@EHGNs (**b**), corresponding line chart of b (**c**), dose-response curve from FL signals with AFP different AFP concentrations (**d**) together with corresponding detection range of d ((**e**), the inset illustrates the calibration curve) and dose-response curve from UV values with different AFP concentrations (**f**) as well as the corresponding detection range of f (**g**).

Cysteine is immediately immobilized on the freshly prepared EHGNs surface (Supplementary Figure S1) via gold–sulfur bonds to serve as a linker molecule for subsequent antiAFP immobilization via EDC/NHS (mole ratio = 3:1) activation to achieve CySiQDs@EHGN and antiAFP/SiQDs@EHGN nanocomposites (Figure 4a). Preliminary experiments indicated that, given the available sites of antibody on antiAFP/SiQDs@EHGNs, the capture antibody (antiAFP) of 1.0 µg/mL is preferred. An incubating time of 60 min under room temperature is enough for the preparation of the immunoassay probe (Figure 4b,c). On the other hand, a different matrix may affect the activity of antibodies [39]. To evaluate the performance of the fluorescent probes, we experimentally conducted matrix effect assessments. By spiking CEA, CA19-9, HSA, milk, and hemolytic serum into the sample extract, and then diluting it to appropriate concentration to react for 30 min for assessment, recognition performance by FL acuitistion was achieved. The results are listed in Supplementary Table S1. All involved cross-reactive substances, including the signal from the blank control, taking high sample recovery rates, were significantly lower than those in the positive samples, demonstrating that the fluorescent probe exhibits excellent reactivity. Subsequently, the immunoassay probe was blocked with 1.0 µg/mL of bovine serum albumin (BSA) to eliminate non-specific adsorption. hen, under the optimized conditions, application of AFP (800.0 ng/mL, 700.00 ng/mL, 600.0 ng/mL, 500.0 ng/mL, 400.0 ng/mL, 300.0 ng/mL, 200.0 ng/mL, 100.0 ng/mL, 50.0 ng/mL, 25.0 ng/mL, 12.5 ng/mL, 6.25 ng/mL, 3.125 ng/mL) to establish dose-responsive relationship . Two independent detection protocols were, respectively, used to evaluate the performance of the constructed immunoassay, using FL and UV–vis signals (Figure 4d,f). The difference in the linear range of the two methods stems from their different detection principles. The changes in the fluorescence signal may be limited by the binding site saturation on the probe’s surface and the energy transfer efficiency, while the LSPR-based UV absorption signal responds more broader to changes in the refractive index of the medium. It is noteworthy that in the common interval of 0.0–400.0 ng/mL of AFP, both methods showed highly consistent good responses (R^2^ = 0.99, Figure 4e,g). Upon comparison, it was found that the fluorescence immunoassay is more suitable for detection scenarios requiring high sensitivity, while the UV immunoassay provides an alternative choice for quantification over a wide concentration range.

### 3.5. Performance Evaluation

#### 3.5.1. Dose–Response, Sensitivity, and Linear Range

The AFP in serum can be utilized for auxiliary diagnosis of HCC, demonstrating significant clinical value [40]. Compared to conventional nanomaterial-based methods that immobilize antibodies on surfaces for electrochemical or radioimmunoassay detection [18,41,42], this method employs nanomanipulation to introduce fluorescent silicon quantum dots into porous gold nanoshells. The retained fluorescence signal provides enough signals for AFP measurement. Two independent detection methods of the constructed immunoassay were used to comprehensively evaluate the performance of FL and UV–vis analyses. FL analysis presents higher sensitivity (Figure 4d). There is a quantitative relationship between AFP concentrations and fluorescence signals. Fluorescence intensity showed a good linear relationship with AFP concentration in the range of 3.125–200.0 ng/mL, with y = 88.6453x + 310.0376 and a correlation coefficient of 0.9919 (Figure 4e). With PBS as the blank, the signal-to-noise ratio is 3, and by calculation, the detection limit (LOD) is 0.234 ng/mL, which can basically meet the needs of clinical testing, as criteria of auxiliary diagnosis typically use 25.0 ng/mL as the threshold value, with persistent levels of 200.0 ng/mL for two months or 400.0 ng/mL for one month, serving as the standard for HCC diagnosing. The detection range of this method demonstrates a slight comparative advantage over the results reported of 20–200.0 ng/mL by immunoassay based on gold nano-mushroom arrays [43] and of 0.5–45 ng/mL using a CdTe-quantum-dot-labeled biosensor [18]. AFP detection by UV values also maintains excellent linear relations in the range of 0.0–400.0 ng/mL (Figure 4f) deduced from equation y = 0.0026x + 1.0264 (R^2^ = 0.99) with a LOD of 10.1 ng/mL (Figure 4g).

#### 3.5.2. Precision

Precision of the proposed method is a key indicator used for appraising its practical application. We examined this by using intra-group and between-group repeatability experiments. Using three concentration levels of 25.0 ng/mL, 50.0 ng/mL, and 100.0 ng/mL of AFP, the deviation in group repeatability (n = 5) was less than 1% (0.50%, 0.22%, and 0.81%, respectively), demonstrating acceptable high precision. The deviation of repeatability between groups was slightly higher (1.55%, 1.04%, and 1.52%, respectively), which achieves the desired outcome of minor changes at different times and between different operating batches. However, the values still fully met the conventional requirements of bioanalysis.

#### 3.5.3. Accuracy

Since SiQDs within the encapsulated porous gold nanoshells are shielded from interference by irrelevant substances without signal attenuation in the detection solution, the assay exhibits excellent reproducibility. After chemical surface immobilization and sealing of antibodies, a detection surface for antigens is formed. Variations in chemical surface properties may lead to differences in antibody activity, thereby affecting antigen-binding efficiency [44]. To compare antibody activity differences, we conducted antigen/antibody reactions using 100.0 μg/mL of anti-AFP and 25.0 ng/mL of AFP to evaluate the matrix effects, confirming the stability of the detection probe developed using SiQD-embedded porous gold nanoshells. The accuracy of the proposed method was evaluated by a spiked recovery experiment. Sequential concentrations of 100.0 ng/mL, 150.0 ng/mL, and 200.0 ng/mL were prepared by the addition of 50.0 ng/mL of AFP standard into 50.0 ng/mL, 100.0 ng/mL, and 150.0 ng/mL AFP solutions. Recovery of samples was 94.1%, 99.9%, and 107.9%, indicating acceptable accuracy, even possessing a comparative advantage with the reported literature [45,46]. Table 1 displays the comparison of performance with different methods to analyze AFP. Compared to the reported investigations, this proposed immunoassay based on SiQDs@EHGNs have relative lower LODs and an acceptable linear range. More importantly, this proposed method has merits of one-step analysis and simple operation.

#### 3.5.4. Specificity

**Figure 5:**
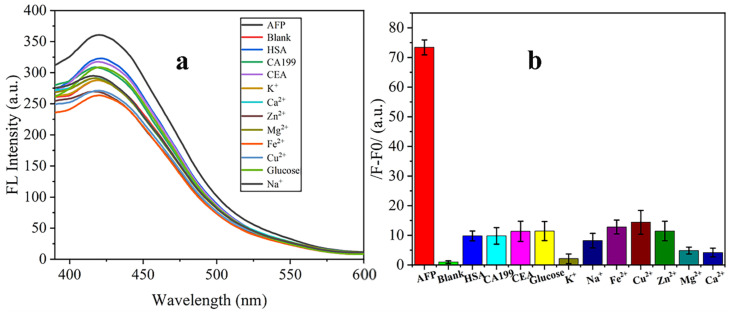
Estimation of specificity of the proposed method by FL intensity measurement (**a**) and net FL signal of different interferents compared with 25.0 ng/mL AFP (**b**).

Specificity of the immunoassay was characterized by evaluating the relative fluorescence signal of antigen and the prepared interference substance in PBS (0.1 M). The fluorescence signals of AFP, blank (PBS, pH 7.2), HAS (1.0 µg/mL), CA199 (1.0 µg/mL), CEA (1.0 µg/mL), glucose (3.9 mM), K^+^ (3.5 mM), Zn^2+^ (62 µM), Mg^2+^ (1.12 µM), Ca^2+^ (2.25 mM), Na^+^ (135 mM), Cu^2+^ (21.8 µM), and Fe^2+^ (7.52 mM) were significantly lower than those of the aforementioned 100.0 ng/mL of AFP. No essential changes in outliers were found for result judgment during the experiment of blank and non-AFP targets, confirming the method’s excellent specificity (Figure 5a,b).

### 3.6. Methodological Comparison

**Figure 6:**
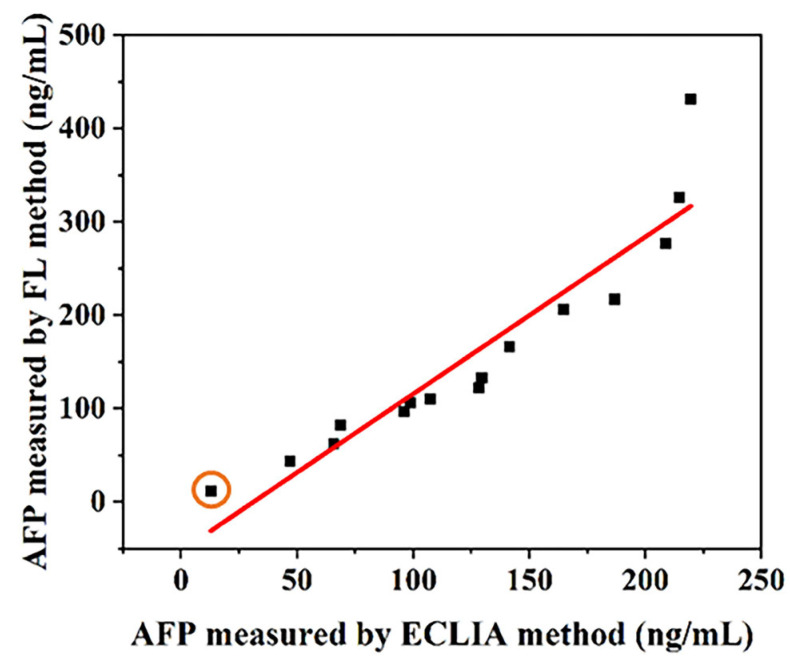
Correlation between conventional ECLIA and FL detection methods was assayed by 15 patient samples. The orange circle-marked data point is derived from serum samples of a healthy patient, while other data points are obtained from patient serum samples.

The detection results of 15 clinic samples using our developed immunoassay method were compared with the results of electrochemiluminescence immunoassay (ECLIA), which is often taken as a gold standard for clinical application, and are shown in Figure 6. There was a high correlation (R = 0.937, *p* < 0.01) by Spearman’s analysis. It has a high agreement for HCC auxiliary diagnosis between two methods. It demonstrated that the proposed method has better potential for serum AFP determination and disease auxiliary diagnosis. In addition, the prepared testing protocol with less time consumption enables one-step AFP detection with simplified operational procedures.

## 4. Conclusions

A one-step immunoassay based on the composite structure of silicon quantum dots and etched hollow gold nanoshells (SiQDs@EHGNs) was successfully constructed, which can realize rapid detection of AFP by dual-mode analysis involving fluorescence signal intensity and ultraviolet signal values. By utilizing the plasma enhancement effect and cavity-loading characteristics of EHGNs, the SiQDs@EHGNs improve the fluorescence stability and signal intensity of SiQDs, and overcome the limitations of signal fluctuation and cumbersome operation steps in traditional fluorescence detection. Under optimal conditions, the method showed a good linear relationship within 3.125–200.0 ng/mL, with a LOD of 0.234 ng/mL (R^2^ = 0.99), and showed good precision, accuracy, and specificity. By contrast, the dynamic range of UV–visible absorption spectroscopy (UV–vis) (0–400 ng/mL) has a detection limit of 10.1 ng/mL (R^2^ = 0.99). This study not only provides an accurate and sensitive analytical tool for early-stage screening of AFP, but it also provides a useful reference for the construction and application of multifunctional nanoprobes in the biosensing field.

## Figures and Tables

**Table 1:** Linear ranges, LODs, and RSDs% of various methods for the detection of AFP.

Reported Methods of AFP Analysis	Linear Range (ng/mL)	LOD (ng/mL)	RSD (%)	Reference
ECLIA	0.2–100	0.0748	4.9–9.8	[7]
PECIA	0.00005–50	0.00015	8.4	[47]
ELISA	20–600	9.7	2.45–3.97	[8]
RFEIA	0.125–8	0.013	0.21–13.8	[48]
ECIA	0.01–20	0.05	3.9	[12]
MSPE-ID-LC-MS/MS	1.0–500.0	1.5	3.0–8.8	[49]
QCM	13–760	9.6	1.8	[50]
LFA	10.0–100.0	7.08	-	[51]
SERS-LFIA	0.001–500.0	0.00072	3.17–6.06	[52]
CySiQDs@EHGNs	3.125–200.0	0.234	0.22–1.55	This study

## Data Availability

The original contributions presented in this study are included in the article/Supplementary Materials. Further inquiries can be directed to the corresponding authors.

## Supplementary Materials

The following supporting information can be downloaded at: https://www.mdpi.com/article/10.3390/nano16080479/s1, Table S1. Matrix effect of the proposed method appraised by recovery rates. Figure S1. UV-vis absorbance spectra of CySiQDs@EHGNs prepared by different concentrations cysteine immobilized on SiQDs@EHGNs surface (a) and their corresponding potentials.

## References

[1] Bray F., Ferlay J., Soerjomataram I., Siegel R.L., Torre L.A., Jemal A. (2018). Global Cancer Statistics 2018: GLOBOCAN Estimates of Incidence and Mortality Worldwide for 36 Cancers in 185 Countries. CA A Cancer J. Clin..

[2] Balogh J., Victor D., Asham E.H., Burroughs S.G., Boktour M., Saharia A., Li X., Ghobrial R.M., Monsour H.P. (2016). Hepatocellular carcinoma: A review. J. Hepatocell. Carcinoma.

[3] Galle P.R., Foerster F., Kudo M., Chan S.L., Llovet J.M., Qin S., Schelman W.R., Chintharlapalli S., Abada P.B., Sherman M. (2019). Biology and significance of alpha-fetoprotein in hepatocellular carcinoma. Liver Int..

[4] Bergstrand C.G., Czar B. (1956). Demonstration of a new protein fraction in serum from the human fetus. Scand. J. Clin. Lab. Investig..

[5] Dai Y., Cai Y., Zhao Y., Wu D., Liu B., Li R., Yang M., Wei Q., Du B., Li H. (2011). Sensitive sandwich electrochemical immunosensor for alpha fetoprotein based on prussian blue modified hydroxyapatite. Biosens. Bioelectron..

[6] Sauzay C., Petit A., Bourgeois A.-M., Barbare J.-C., Chauffert B., Galmiche A., Houessinon A. (2016). Alpha-foetoprotein (AFP): A multi-purpose marker in hepatocellular carcinoma. Clin. Chim. Acta.

[7] Yu Z., Huang L., Chen J., Li M., Tang D. (2021). Graded oxygen-doped CdS electrode for portable photoelectrochemical immunoassay of alpha-fetoprotein coupling with a digital multimeter readout. Sensors Actuators B Chem..

[8] Liu J., Jiang Y., Chen X., Chen L., Zhang X., Cui D., Li Y., Liu Z., Zhao Q., Diao A. (2022). Development of active affibody aggregates induced by a self-assembling peptide for high sensitive detection of alpha-fetoprotein. Chem. Eng. J..

[9] Kemp H.A., Simpson J.S., Woodhead J.S. (1981). Automated two-site immunoradiometric assay of human alpha-fetoprotein in maternal serum. Clin. Chem..

[10] Yamagata Y., Katoh H., Nakamura K., Tanaka T., Satomura S., Matsuura S. (1998). Determination of α-fetoprotein concentration based on liquid-phase binding assay using anion exchange chromatography and sulfated peptide introduced antibody. J. Immunol. Methods.

[11] Chen C., Zhao J., Lu Y., Sun J., Yang X. (2018). Fluorescence immunoassay based on the phosphate-triggered fluorescence turn-on detection of alkaline phosphatase. Anal. Chem..

[12] Zhou J., Zhang C., Chen Y., Wang Z., Lan L., Wang Y., Han B., Pan M., Jiao J., Chen Q. (2019). A simple immunosensor for alpha-fetoprotein determination based on gold nanoparticles-dextran-reduced graphene oxide. J. Electroanal. Chem..

[13] Wang Y.-W., Chen L., Liang M., Xu H., Tang S., Yang H.-H., Song H. (2017). Sensitive fluorescence immunoassay of alpha-fetoprotein through copper ions modulated growth of quantum dots in-situ. Sensors Actuators B Chem..

[14] Du L., Li Z., Yao J., Wen G., Dong C., Li H.-W. (2019). Enzyme free glucose sensing by amino-functionalized silicon quantum dot. Spectrochim. Acta Part A Mol. Biomol. Spectrosc..

[15] Ounkaew A., Antoniuk D., Veinot J.G.C., Bouvier M., Constantinescu I., Tees-DeBeyer W., Hung S., Kizhakkedathu J.N., Narain R. (2025). Biocompatible Water-Soluble Silicon Quantum Dots for Photodynamic Cancer Therapy. ACS Appl. Nano Mater..

[16] Li D., Xu X., Zhou P., Huang Y., Feng Y., Gu Y., Wang M., Liu Y. (2019). A facile synthesis of hybrid silicon quantum dots and fluorescent detection of bovine hemoglobin. New J. Chem..

[17] Morozova S., Alikina M., Vinogradov A., Pagliaro M. (2020). Silicon quantum dots: Synthesis, encapsulation, and application in light-emitting diodes. Front. Chem..

[18] Zhou L., Ji F., Zhang T., Wang F., Li Y., Yu Z., Jin X., Ruan B. (2019). An fluorescent aptasensor for sensitive detection of tumor marker based on the FRET of a sandwich structured QDs-AFP-AuNPs. Talanta.

[19] Sreejith S., Ajayan J., Radhika J.M., Reddy N.V.U., Manikandan M., Mathew J.K. (2024). Recent Progress in Silicon Quantum Dots Sensors: A Review. Silicon.

[20] Ning X., Mao C., Zhang J., Zhao L. (2022). Fluorescence sensing of chloramphenicol based on oxidized single-walled carbon nanohorn/silicon quantum dots- aptamers. J. Mol. Struct..

[21] Hu X., Zhang X., Cao H., Huang Y. (2022). Cu-based metal-organic frameworks-derived copper nanoclusters with tunable emission for ratiometric pH sensing. Sensors Actuators B Chem..

[22] Topete A., Varela A., Navarro-Real M., Rial R., Pardo A., Taboada P. (2025). Revisiting gold nanoshells as multifunctional biomedical nanotools. Coord. Chem. Rev..

[23] Ling J., Zhang Z., Zhang W., Wen D., Ding Y. (2024). Ultra-sensitive fluorescent detection of strychnine based on carbon dot self-assembled gold nanocage sensing probe. Anal. Methods.

[24] Huang C., Wang B., Chen Y., Jin G. (2020). Label-Free Immunoassay of Carcinoembryonic Antigen by Microfluidic Channel Biosensor Based on Imaging Ellipsometry and Its Clinic Application. Integr. Ferroelectr..

[25] Huang C., Tang H., Chen H., Huang X., Fang Y., Wang K., Men R., Gao J., Wang Y., Chen Y. (2022). Comparison of Random and Site-Directed Immobilization of Antibody for α-Fetoprotein Direct Immunoassay by Protein Chip. Integr. Ferroelectr..

[26] Yang K., Yang F., Lu X., Li H., Yang Z., Yin Q., Zhang L., Long Y., Shen C., Chen L. (2025). Facile Immunoassay Constructed by Gold Nanostar-Labeled Rabbit-AFP Antibody and Gold Nanoparticle-Conjugated Goat Anti-Rabbit IgG. Nanomaterials.

[27] Guan Y., Xue Z., Liang J., Huang Z., Yang W. (2016). One-pot synthesis of size-tunable hollow gold nanoshells via APTES-in-water suspension. Colloids Surf. A Physicochem. Eng. Asp..

[28] Cui F., Sui Y., Zhang Y., Xie Z., Yan J. (2023). Silica Etching without HF in Particle Brushes. ACS Chem. Health Saf..

[29] Monk D.J., Soane D.S., Howe R.T. (1993). Determination of the Etching Kinetics for the Hydrofluoric Acid/Silicon Dioxide System. J. Electrochem. Soc..

[30] Chi Y., Sun W., Zhou L., Pei S., Zeng H., Cheng Y., Chai S. (2023). The preparation of hybrid silicon quantum dots by one-step synthesis for tetracycline detection and antibacterial applications. Anal. Methods.

[31] Kadian S., Manik G., Das N., Nehra P., Chauhan R.P., Roy P. (2020). Synthesis, characterization and investigation of synergistic antibacterial activity and cell viability of silver–sulfur doped graphene quantum dot (Ag@S-GQDs) nanocomposites. J. Mater. Chem. B.

[32] Chai S., Chi Y., Sun W., Pei X., Pei S., Sun C., Luo K., Yao B. (2024). Bifunctional Silicon Quantum Dots for Antibacterial Application and Highly Sensitive Detection of Tetracycline. J. Anal. Test..

[33] Tan H., Iso Y., Isobe T. (2025). Photoluminescence enhancement of 1,2,4-triaminobenzene molecules by modification with hydrolytically polycondensed 3-aminopropyltriethoxysilane. J. Lumin..

[34] Peña-Alonso R., Rubio F., Rubio J., Oteo J.L. (2007). Study of the hydrolysis and condensation of γ-Aminopropyltriethoxysilane by FT-IR spectroscopy. J. Mater. Sci..

[35] Hong C., Wu S., Chen S., Lin Z., Chen Q. (2025). Highly sensitive colorimetric method for the detection of organophosphorus pesticides based on H2O2 etching of silver-coated gold nanostars. Sensors Actuators B Chem..

[36] Zhu J., Bart-Smith H., Begley M.R., Zangari G., Reed M.L. (2009). Formation of Silicon Nanoporous Structures Induced by Colloidal Gold Nanoparticles in HF/H_2_O_2_ Solutions. Chem. Mater..

[37] Kim B.S., Chen Y.-T., Srinoi P., Marquez M.D., Lee T.R. (2019). Hydrogel-Encapsulated Mesoporous Silica-Coated Gold Nanoshells for Smart Drug Delivery. Int. J. Mol. Sci..

[38] Jeong S., Kim M.-W., Jo Y.-R., Kim N.-Y., Kang D., Lee S.Y., Yim S.-Y., Kim B.-J., Kim J.H. (2019). Hollow Porous Gold Nanoshells with Controlled Nanojunctions for Highly Tunable Plasmon Resonances and Intense Field Enhancements for Surface-Enhanced Raman Scattering. ACS Appl. Mater. Interfaces.

[39] Musso G., Zoccarato M., Gallo N., Plebani M., Basso D. (2024). Hook-effect in MAGLUMI immunoassay for serum anti-GAD antibodies in neurological disorders: When “wrong” matrix is the right choice. Clin. Chim. Acta.

[40] Niaki N.M., Hatefnia F., Heidari M.M., Tabean M., Mobed A. (2025). Alpha-Fetoprotein (AFP) biosensors. Clin. Chim. Acta.

[41] Yang C., Zhou Y., Fan C., Wang H., Xu J., Wu Z., Song Y., Hu Y., Tian M., Liu G. (2025). Gold nanotriangle-enhanced high-sensitive determination of alpha-fetoprotein via lateral flow immunoassay in serum. Microchim. Acta.

[42] Xu X., Ying Y., Li Y. (2012). One-step and label-free detection of alpha-fetoprotein based on aggregation of gold nanorods. Sensors Actuators B Chem..

[43] Li W., Jiang X., Xue J., Zhou Z., Zhou J. (2015). Antibody modified gold nano-mushroom arrays for rapid detection of alpha-fetoprotein. Biosens. Bioelectron..

[44] Liu Y., Cheng L., Lin S., Yang Y., He Y., Su C., Chen J., Lin Z., Hong G. (2025). Simple and rapid multicolor sensor for seminal plasma ROS detection based on synergistic catalytic etching of gold nanobipyramids dopped agarose composite gel. Talanta.

[45] Li J., Jiang Y., Xu A., Luo F., Lin C., Qiu B., Lin Z., Jiang Z., Wang J. (2024). ZnO/Au/GaN heterojunction-based self-powered photoelectrochemical Sensor for alpha-fetoprotein detection. Talanta.

[46] Lou F., Wang S., Han B., Li Q., Tang D. (2024). Portable photoelectrochemical immunoassay with micro-electro-mechanical-system for alpha-fetoprotein in hepatocellular carcinoma. Anal. Chim. Acta.

[47] Li L., Weng Y., Sun C., Peng Y.-Y., Xie Q. (2023). Photoelectrochemical immunoassay of alpha-fetoprotein based on a SnO_2_/In_2_S_3_ heterojunction and an enzyme-catalyzed precipitation strategy. Sensors Diagn..

[48] Su B., Bei Z., Pei H., Xie X., Sun Z., Chen Q., Cao H., Liu X. (2022). Generation of a nanobody-alkaline phosphatase heptamer fusion for ratiometric fluorescence immunodetection of trace alpha fetoprotein in serum. Int. J. Biol. Macromol..

[49] Sun H., Liu J., Zhang Q., Yang L., Song D., Zhou M., Song D. (2026). Development of an SI-traceable magnetic solid-phase extraction coupled with isotope dilution LC-MS/MS method for accurate quantification of alpha-fetoprotein in human serum. Talanta.

[50] Chen Y., Shi H., Mu B. (2023). Application of a Novel One-Side Cell Quartz Crystal Microbalance Immunosensor in the Determination of Alpha-Fetoprotein from Human Serum. Diagnostics.

[51] Ma M., Zhang M., Wang J., Zhou Y., Zhang X., Liu G. (2025). Rapid Detection of Alpha-Fetoprotein (AFP) with Lateral Flow Aptasensor. Molecules.

[52] Li J., Fang C., Yao Y., Chen L., Lin B., Wang Y., Guo L. (2024). Rapid antibody conjugation strategy via instant charge inversion of AuNBPs toward ultrasensitive SERS-LFIA detection of AFP. Microchem. J..

