# Supplementary material for: One-Step Immunoassay of Alpha-Fetoprotein Constructed by Silicon-Quantum-Dot-Loaded Porous Gold Nanoshells
Source: Nanomaterials (Basel). 2026 Apr 17;16(8):479. doi: 10.3390/nano16080479 (PMC13118350; doi:10.3390/nano16080479)
Supplement: Supplementary file 1 [file nanomaterials-16-00479-s001.zip › nanomaterials-4245004-supplementary.pdf]

Supplement materials

Table S1. Matrix effect of the proposed method appraised by recovery rates.

Matrix types	Spiked C.(ng/mL)	measured C.(ng/mL)	Recovery rate (%)
HSA(1.0μg/mL)	50	48.2	96.4
	100	97.5	97.5
	150	147.2	98.1
HSA (5.0μg/mL)	50	49.5	99.0
	100	101.8	101.8
	150	152.5	101.7
HSA (10.0μg/mL)	50	51.2	102.4
	100	103.6	103.6
	150	154.8	103.2
Milk(1%)	50	46.5	93.0
	100	94.8	94.8
	150	143.5	95.7
Milk(5%)	50	44.2	88.4
	100	89.5	89.5
	150	136.5	91.0
Milk(10%)	50	47.8	95.6
	100	96.5	96.5
	150	145.8	97.2
Mild hemolytic serum	50	48.8	97.6
	100	98.2	98.2
	150	148.5	99.0
Severe hemolytic serum	50	46.5	93.0
	100	94.2	94.2
	150	142.8	95.2

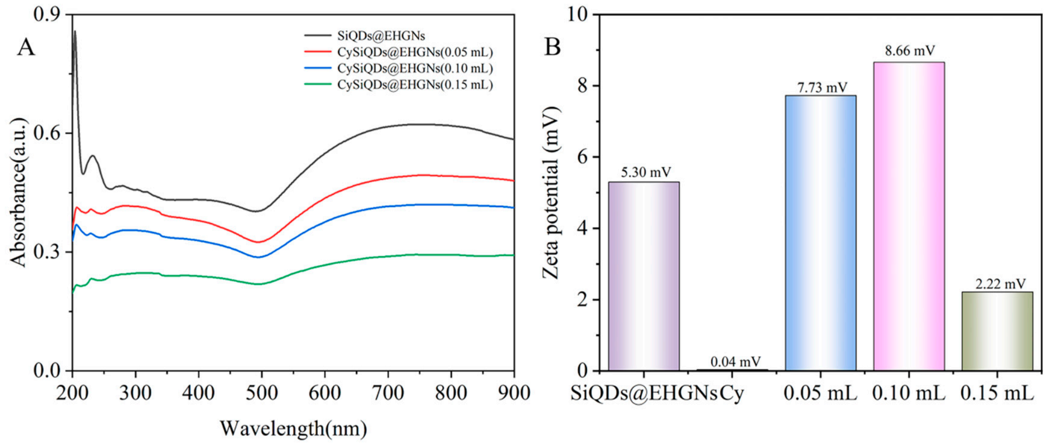

Figure S1. UV-vis absorbance spectra of CySiQDs@EHGNs prepared by different concentrations cysteine immobilized on SiQDs@EHGNs surface (a) and their corresponding potentials.
